# Searching for meaning is associated with costly prosociality

**DOI:** 10.1371/journal.pone.0258769

**Published:** 2021-10-25

**Authors:** Brodie C. Dakin, Simon M. Laham, Nicholas Poh-Jie Tan, Brock Bastian

**Affiliations:** Melbourne School of Psychological Sciences, University of Melbourne, Melbourne, Australia; Coventry University, UNITED KINGDOM

## Abstract

The study of meaning in life has largely centered on its relationship with personal well-being, while a focus on how meaning is related to enhancing the well-being of others has received less research attention. Although searching for meaning may imply lower personal well-being, we find that meaning-seekers are more motivated to perform costly prosocial actions for the sake of others’ well-being, given the perceived meaningfulness of these behaviors. Studies 1–4 (*N* = 780) show that meaning-seeking correlates with the motivation to engage in a range of costly prosocial behaviors. Meaning-seeking is further shown to be distinct from pursuing happiness in its relationship with costly prosociality (Study 2 & 3) and to share a stronger association with high-cost than low-cost prosociality (Study 3 & 4). Study 5 (*N* = 370; pre-registered) further shows that the search for meaning is related to costly prosocial behavior in the recent past. While our studies are cross-sectional, the pattern of findings suggests that seeking meaning (rather than happiness) may play an important role in motivating altruistic tendencies.

## Introduction

Meaning in life has predominantly been studied in terms of how it influences personal well-being, or *feeling good* [1]. Less work has examined how meaning in life is related to increasing the well-being of others, or *doing good*. Prior research has revealed that people draw meaning from prosocial behavior [2, 3], suggesting that prosociality may offer utility for those who are searching for meaning in life. While previous work has highlighted the downsides of meaning-seeking, such as reduced personal well-being, it may be that people searching for meaning are more motivated to perform behaviors that promote social good. Compared to those primarily searching for happiness, meaning-seekers may be more willing to bear the personal costs associated with prosocial acts, given that the burden of helping others may be a powerful source of existential meaning.

## Meaning in life and the search for meaning

Meaning in life comprises one’s perception of the coherence (sense of clarity), purpose (goals and aims) and significance (sense of mattering) in one’s own life [4, 5]. The importance of meaning in life within the psychological literature largely rests on its recognition as a “cornerstone” of well-being [6]. Possessing meaning shares a strong relationship with life satisfaction, happiness, and positive affect [1, 7], and serves as a kind of bulwark against the stressors inherent to living [8]. Theorists have argued that people generally develop a sense of meaning by engaging in ‘self-transcendent’ pursuits [9, 10], or connecting oneself, through goals, values, and behavior to things that are *external to* and *greater than* the self. This is evidenced in empirical work, which shows that many sources of meaning in life involve connecting to things external to the self (e.g., relationships, religion, societal institutions, etc.) [11, 12].

Since the publication of the Meaning in Life Questionnaire (MLQ) by Steger and colleagues [1], the study of meaning has largely centered on two constructs (represented by the two subscales comprising the MLQ): presence of meaning and search for meaning. Presence of meaning captures the extent to which a person feels that their life is satisfyingly meaningful. Several studies indicate that presence of meaning is associated with a host of positively-valenced personal well-being outcomes, such as life satisfaction, positive affect, and psychological need satisfaction, as well as decreased depression, anxiety, and neuroticism [1, 7, 13].

Search for meaning represents one’s need to actively establish a greater sense of meaningfulness in one’s life. While search and presence are not opposites, search for meaning differs from presence through its association with poorer personal well-being. Previous work has shown that search for meaning is correlated with increased neuroticism, sadness, and depression, increased rumination, and a reduced sense of relatedness, self-acceptance, and life satisfaction [1, 13]. From a personal well-being perspective, searching for meaning fits a more deficit-based account, particularly for Westerners [14].

Moving away from a focus on well-being, *Significance Quest Theory* (SQT) suggests that meaning-seeking may be associated with engagement in socio-political activities [15]. For instance, studies have found that search for meaning relates to endorsing extreme political orientations [16], ideological self-sacrifice, and violent extremism [17–19]. Search for meaning is argued to represent a quest for personal significance [15], but this ‘quest’ has generally only been examined in the context of extreme, anti-social, or strictly ideological behavior.

In contrast to accounts which associate meaning-seeking with poorer well-being and anti-social behavior, there is also reason to think that searching for meaning in life may lead people to promote social good. Those seeking meaning may be more willing to make the personal sacrifices implicit within prosocial acts, because these acts are themselves powerful sources of meaning.

## Prosociality as a powerful source of meaning

People derive meaning from a variety of sources in life, such as social belonging [20], spirituality [21], or from their work [22]. While there are myriad sources of meaning, one of the more established ways that people find meaning is through prosocial actions [23–25]. Evidence that prosociality increases perceptions of meaning has been found in several empirical studies. Aspects of prosociality such as being a ‘giver’ and volunteering are correlated with having a stronger sense of meaning [26–28], and volunteering is also associated with increases in meaning over time [29]. Prosociality and meaning are also related at the daily level [30–32], with evidence showing that performing prosocial acts in everyday life is associated with experiencing greater meaning on those days [33, 34]. Finally, experimental studies have found that performing prosocial actions, such as writing letters of gratitude [3], and spending money to benefit others [2] enhance the actor’s sense of meaning. Making a prosocial impact has been shown to feel meaningful even when no contact is made with the recipient [35].

In sum, evidence suggests that the meaningfulness of prosocial action may make these behaviors attractive to those searching for meaning in life. If searching for meaning motivates behaviors which enhance one’s sense that their life matters and has purpose, then it follows that meaning-seekers should show an inclination toward prosocial behavior, as these behaviors lead to experiencing greater meaning. The reason *why* prosocial behavior is a source of meaning may come down to several factors. First, prosocial acts may increase one’s sense of meaning because they usually result in increased social connection [3], which in turn facilitates meaning [20]. Second, prosocial acts are closely tied to cultural values (e.g., Benevolence and Universalism) [36], and acting out values is associated with experiencing meaning [37]. However, a third element that may be critical in making prosocial behavior meaningful is the *costliness* (i.e., inherent elements of pain, effort, and/or resource-intensiveness) inherent in these behaviors.

## The role of cost in making prosociality meaningful

Generally, it is assumed that humans are fundamentally motivated to maximize their hedonic experience by seeking pleasure and avoiding pain [38]. However, costly behaviors can have utility, with research showing that experiencing pain, effort, or other costs while enacting a behavior may increase both the perceived value of the action, and the value of what was sacrificed for [39, 40] (see also ’effort justification’). This relationship between costliness and valuing has been demonstrated in several studies [41–45].

Furthering this previous work, Olivola and Shafir [43, 44] provide evidence that the meaningfulness of prosocial endeavors is at least partially determined by the costliness of the act. Specifically, they found that when a charitable fundraiser requires a costly effort (i.e., running several miles), participants offered to donate more money, and ascribed greater meaning to the fundraiser, compared to when it involved a less costly effort (i.e., a picnic). This finding is further supported by work on the construct of ‘heroism’, where the perceived meaningfulness of heroic acts [46] may be driven by the costliness of these behaviors [47].

While prosocial acts may be meaningful for several reasons, these findings indicate that the costliness of prosocial acts is important in determining their meaningfulness. Thus, because *costly* prosocial acts tend to be viewed as sources of meaning, those who are searching for meaning may be especially drawn toward performing these types of behaviors. This link between meaning-seeking and costly prosociality has already seen some support by Igou and colleagues [48], who found that search for meaning mediated the relationship between experiencing regret and motivation to act heroically. In essence, seeking meaning may put an individual in a unique motivational stance where they are driven to enact behaviors which are both costly for themselves and beneficial for others. This motivation to perform meaningful acts that are often costly and hedonically-negative creates a tension between the motivation to pursue meaning in life, and the motivation to pursue states of happiness.

## The search for meaning versus the pursuit of happiness

While the motivation to achieve meaning and happiness in life are correlated [49], a tension may exist between *pursuing* positive-hedonic experiences versus performing acts which establish meaning. While the *experiences* of happiness and meaning in life are strongly related [1, 7] there can be differences in what is associated with feeling happy versus a sense of meaning [26, 27]. For example, Choi and colleagues [33] found in an experience-sampling study that performing pleasure-oriented behaviors (e.g., watching TV, attending a party) was positively associated with momentary happiness but negatively with momentary meaning. Conversely, more effortful behaviors (e.g., visiting a hospital, work) were associated positively with meaning but negatively with happiness. Further, unlike happiness, meaning can be derived from hedonically-negative experiences [34, 50, 51].

This pattern of results suggests that happiness may be more associated with leisure and self-orientation, while the experience of meaning is more associated with difficult experiences and concern for the needs of others. While some research has shown prosocial behavior can increases happiness [52, 53], the relationship between prosociality and happiness is small [54], and not as strong as the relationship with meaning [33]. Thus, it is less likely that a focus on obtaining positive hedonic states would motivate people to engage in costly prosocial acts.

The differences between the *experience* of meaning and happiness may in turn inform differences between the *pursuit* of meaning and happiness. Specifically, while searching for meaning may relate to costly or negative-hedonic behaviors performed to benefit others, pursuing happiness alone would be unlikely to motivate these behaviors. Huta and Ryan [49] have provided indirect support for this, showing that pursuing happiness is related with “carefreeness”. This indicates that pursuing happiness alone may discourage taking on the concerns of others, particularly when doing so is costly for the self. Therefore, in contrast with the search for meaning, pursuing happiness is likely negatively related or unrelated to costly prosociality.

## Overview of present research

Five studies tested whether people’s search for meaning is related to costly prosociality (voluntary engagement in behaviors that involve pain, effort and/or resource expenditure performed to enhance the well-being of others). Study 1 examined the relationship between the search for meaning and the motivation to enact costly prosocial behaviors. Study 2 examined whether the pursuit of happiness is differently related to costly prosocial behavior motivation compared to the search for meaning. Study 3 again compared the search for meaning and pursuit of happiness, and additionally tested if meaning-seeking predicts motivation toward high-cost versus low-cost prosociality differently. Study 4 sought to replicate the search for meaning—costly prosociality relationship while controlling for personality dimensions (Agreeableness and Open-mindedness) which have been linked with meaning-seeking and prosociality. Finally, Study 5 extended previous studies that examined prosocial *intention* by considering how the search for meaning is related to reported engagement in everyday costly prosocial behaviors.

## Data collection, analysis, and availability

All studies were approved by the Human Ethics Advisory Group at the University of Melbourne (Ethics approval number: 1851599.1). Data from all studies was collected between December 2017 and January 2019 on Amazon Mechanical Turk (MTurk) using American participants. To qualify for participation, MTurk workers had to possess an approval rating of >95% and have had completed at least 100 HITs previously. In addition to this selection criteria, we removed participant cases across the studies who completed the survey in less than one-third of the median completion time (Studies 1–5), failed any attention check measures (Studies 2–5) or produced a suspect response on a ‘qualitative’ measure (Studies 2–4). A full description of our attention checks, data exclusion procedure, and indicators of data quality are included in the supporting information (S1 File). Data analysis was performed in Rstudio (version 1.4.1103) using the *tidyverse* and *psych* packages. Materials, de-identified data, and analysis scripts for all studies are available on the Open Science Framework (https://osf.io/hrp78/).

## Study 1

Study 1 tested the relationship between search for meaning and motivation to enact a range of costly prosocial behaviors. Following from our argument, we hypothesized that the search for meaning would be positively related to costly prosociality.

### Method

#### Participants and procedure

To ensure our study had a sample size which would allow for adequate statistical power, we performed a power analysis on G*Power (version 3.0). Expecting a small-to-medium correlation size (*r* = .20), and setting power at .80, the analysis revealed that a sample of 193 participants would be required for the study to be sufficiently powered. In anticipation of having to remove some participant data, we aimed to recruit 200 participants. Two-hundred and ten complete responses were received for the study on Amazon’s Mechanical Turk (MTurk) by American participants who received (US) $1.00 payment. We removed 14 participant responses who completed the study in less than one-third of the median completion time, resulting in a final sample of 196 participants (111 male, 85 female) aged between 18 and 76 years (*M*_*Age*_ = 35.30, *SD*_*Age*_ = 10.80). Participants first completed the MLQ, then costly prosociality measures, and lastly rated how meaningful they believed self-sacrificing for others was and filled out demographic questions.

#### Measures

*Meaning in life questionnaire*. The MLQ was used to measure search for meaning and presence of meaning [1]. Example search items include *“I am looking for something that makes my life feel meaningful”* and *“I am seeking a purpose or mission for my life”* (5 items; α = .96). Example presence items include *“I understand my life’s meaning”* and *“I have discovered a satisfying life purpose”* (5 items; α = .95). Participants indicated their agreement with each item on a 7-point Likert scale (*1 = absolutely untrue; 7 = absolutely true*).

Across all studies (excluding Study 4), we included presence of meaning in analyses as a co-variate. While they are not opposites, search for meaning and presence of meaning are generally negatively correlated [1]. Furthermore, some studies have reported that presence of meaning correlates with having greater concern for others [55–57], and so presence of meaning may also be related to the motivation to perform prosocial actions. Therefore, to avoid presence of meaning suppressing the relationship between search for meaning and prosociality, we included it as a co-variate where appropriate.

*Costly prosocial behavior measure 1*: *Kidney donation*. To measure inclination toward costly prosocial behavior, participants were asked to imagine a scenario in which a local hospital had a plethora of patients in desperate need of a kidney transplant [58]. Participants were asked how likely they would be to donate one of their kidneys (assuming they had both) to each of the four potential recipients: 1) family member, 2) work colleague, 3) welfare recipient, and 4) charity worker. Likelihood of kidney donation in the case of each recipient was indicated separately on a 7-point Likert scale (*1 = extremely unlikely; 7 = extremely likely*). Given the good reliability of the four items (α = .84), an aggregate ‘kidney donation’ measure was used for analyses.

*Costly prosocial behavior measure 2*: *Self-sacrifice for entities*. *A* second costly prosocial measure gauged willingness to sacrifice one’s own life to save a group of entities [58]. Participants were asked to imagine a scenario where a ruthless dictator was about to wipe out a particular group of entities, but that if they sacrificed their own life, the entity group would be spared. Participants were then presented with four different groups of entities: “people from your hometown”, “people from Africa”, “chimpanzees”, and “redwood trees”. Participants were asked to elect on a 10-point scale how many of each entity would need to be annihilated before they would sacrifice their own life in their place (1 = *1–10*, 2 = *10 to 100*, 3 = *100 to 1000*, 4 = *10%*, 5 = *20%*, 6 = *50%*, 7 = *75%*, 8 = *90%*, 10 = *I would never sacrifice myself*). Individual responses regarding each entity group showed good reliability (α = .81), so an aggregate ‘self-sacrifice for entities’ measure was used for analyses.

*Other measures*. To better gauge our sample’s demographic, we had participants indicate their political ideology regarding both i) economic issues (e.g., social welfare), and ii) social issues (e.g., immigration) on a 7-point scale (1 = *left/liberal;* 7 = *right/conservative*). Further, to test our assumption that self-sacrifice is a meaningful behavior, we asked participants to rate how “meaningful” they think it is to sacrifice one’s life for another entity on an 11-point scale (0 = *not at all meaningful;* 10 = *extremely meaningful*).

### Results and discussion

Participants generally considered self-sacrificing for another entity to be a meaningful behavior (*M* = 6.71, *median* = 8, *SD* = 3.23) and those searching for meaning were marginally more likely to endorse self-sacrifice as meaningful (*r* = .13, *p* = .078).

Table 1 displays zero-order correlations between all primary variables included in the study. Zero-order correlations revealed that search for meaning was significantly negatively related to both presence of meaning and age. Regarding our hypothesis, correlations further reveal that search for meaning was significantly positively related to both costly prosocial measures of self-sacrifice for entities and kidney donation.

**Table 1:** Correlations matrix and descriptives in Study 1.

Variable	*M* (*SD*)	1	2	3	4	5	6	7	8
1. Search for meaning	4.11 (1.76)								
2. Presence of meaning	4.86 (1.57)	**-.43** **							
3. Self-sacrifice (entities)	3.64 (2.46)	**.14** *	.02						
4. Kidney donation	4.18 (1.44)	**.16** *	.11	**.30** **					
5. Meaningfulness S-S	6.71 (3.23)	.13	.08	**.35** **	**.14** *				
6. Age	-	**-.28** **	**.14** *	-.02	-.04	-.03			
7. Female	-	-.08	**.15** *	.06	.07	.07	.11		
8. Conservatism (economic)	3.76 (1.79)	-.12	.12	.01	-.13	-.01	.04	-.11	
9. Conservatism (social)	3.37 (1.94)	-.12	.11	-.00	-.11	-.05	-.02	-.09	**.82** **

*N* = 196

* = *p* < .05

** = *p* < .01; Meaningfulness S-S = perceived meaningfulness of self-sacrificing to save another entity; Self-sacrifice (entities) scores were reversed so that a higher score indicates *greater* willingness to self-sacrifice.

Next, we performed multiple regressions predicting each costly prosocial measure with search for meaning while simultaneously including presence of meaning, age, gender, and political ideology (economic and social) as co-variates (Table 2). Results from these regressions reveal that the search for meaning significantly predicted both measures of costly prosociality (βs = .19-.25, *p*s < .02), with effect sizes growing in strength compared to the zero-order level. The regressions also reveal that presence of meaning was significantly associated with willingness to donate a kidney, but not self-sacrificing for a range of entities. In sum, results from these regressions support our hypothesis that the search for meaning predicts motivation to enact costly prosocial behaviors.

**Table 2:** Multiple regressions predicting costly prosociality with search for meaning and other variables in Study 1.

Variable	Kidney donation		Self-sacrifice (for entities)	
	*β*	*95% CI*	*β*	*95% CI*
(Intercept)	.00	-.14, .14	.00	-.14, .14
Search for meaning	.25**	.09, .40	.19*	.03, .35
Presence of meaning	.23**	.07, .38	.09	-.07, .25
Age	-.01	-.15, .14	.00	-.14, .16
Female	.05	-.09, .19	.06	-.08, .21
Conservatism (economic)	-.12	-.36, .13	.04	-.21, .29
Conservatism (social)	-.01	-.25, .24	-.02	-.27, .23

*N* = 196; β = standardized beta; CI = confidence interval

* = *p* < .05

** = *p* < .01; *R*^2^: Kidney donation = .08, Self-sacrifice = .03.

The stronger relationship between search for meaning and costly prosociality when controlling for co-variates indicated a suppression effect. To investigate this further, we examined partial correlations between search for meaning and costly prosociality, while controlling for each co-variate in turn. When presence of meaning was controlled for, the relationship between search for meaning and costly prosociality increased, indicating that presence of meaning was the variable suppressing the relationship. Conceptually, we interpret this suppression as arising because the more likely a person is to be searching for meaning, the less likely they are to have presence of meaning [1], but being higher in presence of meaning is also somewhat positively related to being prosocial.

## Study 2

Study 2 sought to extend Study 1 by i) replicating the relationship between search for meaning and costly prosociality, and ii) examining whether pursuing happiness has a different relationship to costly prosociality. The motivations to pursue meaning and happiness in life are related [49]. However, compared to meaning, hedonic forms of happiness tend to be more associated with a focus on one’s own needs and level of comfort [26, 33], and are less clearly related to prosociality [2, 33, 54]. As such, we expected that pursuing hedonic forms of happiness would be unrelated or even negatively related to motivation to engage in costly prosocial acts. To mirror controlling for presence of meaning when examining the effects of meaning-seeking, we also measured and controlled for life satisfaction (as an approximation of ‘presence of happiness’) when examining the effect of happiness-seeking.

### Method

#### Participants and procedure

In Study 1, the correlations between search for meaning and costly prosociality fell just short of the effect size anticipated in the previous power analysis. This would suggest recruiting a larger sample for subsequent studies. However, due to budget constraints, Studies 1–4 were limited to recruiting approximately 200 participants each. To address this shortcoming, we chose to include a meta-analysis of the relationship between search for meaning and costly prosociality across Studies 1–5 (see Cross-study meta-analysis). However, we also suggest caution in interpreting results from Studies 1–4 due to sample sizes.

Two hundred and eight American participants completed the study on MTurk in exchange for (US) $1.60 payment. Sixteen cases were removed for either failing an attention check, producing a suspicious response when asked to describe a group they belong to (e.g., writing “good”), or completing the study in less than one-third of the median completion time. This produced a final sample of 191 participants (106 male, 85 female), aged from 21 to 71 years (*M*_*Age*_ = 37.4, *SD*_*Age*_ = 11.0). Participants completed the survey by first filling out the meaning and happiness-relevant scales, then responding to the costly prosociality measures, and finally demographics questions.

#### Measures

Measures of search for meaning (α = .95), presence of meaning (α = .95), kidney donation (α = .82), and self-sacrifice for entities (α = .80) were the same as Study 1. Added measures included a third costly prosociality measure involving a ‘self-sacrifice’ version of the footbridge dilemma, happiness-pursuit measures, and a life satisfaction measure.

*Self-sacrifice in footbridge dilemma*. To increase the range of costly prosociality variables, we included an adapted self-sacrifice version of the footbridge dilemma [59]. In this version, participants were first asked to write down an ‘in-group’ they belonged to (excluding family). They were then presented with a scenario where they were standing on a footbridge, watching below, where a runaway trolley was heading towards five of their in-group members. They were told that if they jumped, they could use their body to stop the trolley, saving their in-group members, but that this would result in their own death. Participants indicated the likelihood that they would jump to sacrifice their life for the group on a 7-point scale (*1 = not at all likely; 7 = extremely likely*).

*Happiness-pursuit*. The pursuit of happiness was operationalized using three measures. The Valuing Happiness Scale (VHS) [60] measured participant’s concern with feeling happy in life (e.g. *“Feeling happy is extremely important to me”*) on a 7-point scale (*1 = strongly disagree; 7 = strongly agree*; 7 items; α = .78). The Prioritizing Positivity Scale (PPS) [61] measured the extent to which participants prioritized happiness when structuring their life (e.g. *“I structure my day to maximize my happiness”*) and was measured on a 9-point scale (*1 = strongly disagree; 9 = strongly agree;* 6 items; α = .89). We also included a ‘search for happiness’ (SFH) scale, which was adapted from the search for meaning subscale by replacing the terms ‘meaning’ and ‘purpose’ with ‘happiness’ and ‘positive emotion’. As with the search for meaning subscale, participants rated the extent to which they were searching for happiness on a 7-point scale (*1 = absolutely untrue; 7 = absolutely true;* 5 items; α = .93). The three happiness-pursuit scales shared moderate-to-strong inter-correlations (*r*s = .30–.64) but did not necessarily factor well together (a Principal Components Analysis revealed that a one-factor solution explained only 42.6% of variance in all happiness-pursuit items). Therefore, we treated each happiness-pursuit variable separate in analyses.

*Life satisfaction*. Life satisfaction was measured with the Satisfaction with Life Scale (SWLS) [62] on a 7-point scale (1 = *strongly disagree*; 7 = *strongly agree*; 5 items; α = .92). The measure was employed to control for the effect of ‘presence of happiness’ when examining the relationship between happiness-pursuit of costly prosociality, as we had controlled for the effect of presence of meaning when examining the search for meaning—costly prosociality relationship.

### Results and discussion

Table 3 displays all correlations between variables included in the study. Search for meaning was positively related to happiness-pursuit measures (*r*s = .27-.41, *p*s < .001), negatively related to life satisfaction, and again negatively related to age and presence of meaning. Presence of meaning was significantly positively related to willingness to donate a kidney, but not the other two costly prosociality measures. Concerning our hypothesis, zero-order correlations revealed that costly prosociality was not significantly related to either search for meaning or happiness-pursuit variables.

**Table 3:** Correlations matrix and descriptives in Study 2.

Variable	*M* (*SD*)	1	2	3	4	5	6	7	8	9	10	11	12
1. Search for meaning	3.96 (1.75)												
2. Presence of meaning	4.85 (1.62)	**-.30** **											
3. Search for happiness	5.57 (1.30)	**.40** **	.07										
4. Valuing happiness	3.89 (1.12)	**.41** **	**-.17** *	**.30** **									
5. Prioritising positivity	6.33 (1.61)	**.27** **	**.27** **	**.64** **	**.49** **								
6. Life satisfaction	4.46 (1.69)	**-.23** **	**.65** **	.03	**-.21** **	**.20** **							
7. Kidney donation	3.98 (1.36)	.04	**.16** *	.08	-.04	.07	**.24** **						
8. Self-sacrifice (entities)	3.08 (2.15)	.13	.04	.00	.04	.06	.11	**.46** **					
9. Self-sacrifice (footbridge)	3.15 (1.98)	.07	.04	-.05	-.07	-.06	.11	**.44** **	**.45** **				
10. Age	-	**-.22** **	.05	-.09	**-.15** *	**-.19** **	-.06	-.02	-.07	**-.18** *			
11. Female	-	.02	-.04	.13	.02	.14	.06	.09	.02	.01	.03		
12. Conservatism (economic)	3.85 (1.86)	-.05	**.23** **	.04	.07	.01	.08	.07	-.02	.04	**.17** *	**-.20** **	
13. Conservatism (social)	3.25 (1.88)	-.10	**.23** **	-.06	.05	-.01	.01	.07	-.08	-.01	**.20** **	**-.16** *	**.75** **

*N* = 191

* = *p* < .05

** = *p* < .01; Self-sacrifice (entities) scores were reversed so that a higher score indicates *greater* willingness to self-sacrifice.

Next, we ran multiple regressions predicting costly prosociality variables with search for meaning and happiness-pursuit variables, while including presence of meaning, life satisfaction, age, gender, and political ideology (economic and social) as co-variates. Results of these regressions (Table 4) revealed that search for meaning was a significant predictor of self-sacrifice for entities, but not kidney donation, or self-sacrifice in the footbridge dilemma. Neither happiness-pursuit variables nor presence of meaning significantly predicted costly prosociality, while life satisfaction predicted kidney donation but not the other costly prosociality measures.

**Table 4:** Multiple regressions predicting costly prosociality with search for meaning in Study 2.

Variable	Kidney donation		Self-sacrifice (for entities)		Self-sacrifice (footbridge)	
	*β*	*95% CI*	*β*	*95% CI*	*β*	*95% CI*
(Intercept)	.00	-.14, .14	.00	-.14, .14	.00	-.14, .14
Search for meaning	.11	-.06, .29	**.19** *	.01, .36	.14	-.04, .31
Presence of meaning	.03	-.18, .24	.02	-.19, .24	.03	-.18, .24
Search for happiness	.07	-.12, .27	-.12	-.32, .08	-.05	-.25, .15
Valuing happiness	-.02	-.21, .16	.00	-.19, .19	-.07	-.26, .11
Prioritizing positivity	-.07	-.30, .16	.06	-.18, .29	-.10	-.33, .13
Life satisfaction	**.25** *	.05, .44	.12	-.07, .32	.10	-.09, .30
Age	-.01	-.16, .14	-.02	-.17, .14	**-.20** *	-.35, -.04
Female	.09	-.06, .23	.01	-.14, .16	.05	-.10, .19
Conservatism (economic)	-.02	-.24, .20	.08	-.15, .31	.13	-.10, .35
Conservatism (social)	.11	-.11, .33	-.13	-.36, .10	-.06	-.28, .17

*N* = 191; *β* = standardized beta; CI = confidence interval

* = *p* <. 05

** = *p* < .01; *R*^2^: Kidney donation = .09, Self-sacrifice (for entities) = .05, Self-sacrifice (footbridge) = .08.

Study 2 intended to replicate the relationship between search for meaning and costly prosociality, and contrast this with pursuing happiness. Regarding our hypothesis, results in Study 2 revealed weaker relationships, with regressions showing that search for meaning significantly predicted one measure of costly prosociality, but not the other two measures. Nonetheless, pursuing happiness was also unrelated to the motivation to enact costly prosocial behaviors.

## Study 3

Study 3 sought to investigate potential reasons for the weaker relationship between search for meaning and costly prosociality found in Study 2. To ensure this result was not an artefact of the measures used, we both altered and expanded our measures of costly prosociality. We also considered that the ‘self-sacrifice for entities’ measure involved an extreme behavior, and produced responses that were not normally distributed, with low mean scores seen in Studies 1 and 2. To address this, we omitted this measure, and included a new sub-scale capturing intentions toward a range of less demanding (albeit still costly) prosocial behaviors, such as volunteering, donating money, and giving blood.

Additionally, we included a sub-scale capturing several low-cost prosocial behaviors. This allowed us to directly test the hypothesis that the search for meaning is particularly related to the motivation to perform *costly*, rather than less costly prosocial acts, given that costly prosocial behaviors are perceived as more meaningful [43, 44]. Finally, we again contrasted meaning-seeking with pursuing happiness as in Study 2.

### Method

#### Participants and procedure

Two-hundred and eight American participants completed the study on MTurk in exchange for (US) $1.85. Thirteen cases were removed following the same exclusion criteria used in Study 2, leaving a final sample of 195 participants (106 male, 88 female, 1 non-binary), aged between 20 and 69 years (*M*_*Age*_ = 34.9, *SD*_*Age*_ = 10.7). Participants completed all meaning and happiness scales before responding to prosociality measures and demographic questions.

#### Measures

Measures of search for meaning (α = .96), presence of meaning (α = .94), search for happiness (α = .94), valuing happiness (α = .83), prioritizing positivity (α = .90), life satisfaction (α = .93), kidney donation (α = .96), and self-sacrifice in the footbridge dilemma were retained from Study 2. The “a family member” kidney donation item was changed to “a distant relative” to lessen the strong negative skew seen for this item in Studies 1 and 2 (skewness = >-2). New measures were as follows.

*High-cost and low-cost prosociality subscales*. We adapted a 12-item prosocial intentions scale, comprising both ‘high-cost’ and ‘low-cost’ prosociality subscales [63]. Participants were asked to indicate their intentions to perform both high-cost prosocial acts (e.g., “*Donate blood regularly (every 3 months)*”; 5 items; α = .77), and low-cost prosocial acts (e.g., “*Donate your unwanted clothes to charity*”; 7 items; α = .82) on a 5-point scale (*1 = not at all likely; 5 = very likely*).

### Results and discussion

Table 5 shows all zero-order correlations between variables included in Study 3. Consistent with previous studies, search for meaning was negatively related to age and presence of meaning and positively related to pursuing happiness. Unlike in Study 2, search for meaning was not significantly related to life satisfaction. Regarding costly prosociality, results provide clear support for our hypothesis, showing that search for meaning was significantly positively related to all measures of costly prosociality (*r*s = .22–.29, *p*s < .003). Further, results show that the search for meaning was a stronger predictor of the high-cost prosociality subscale compared to the low-cost prosociality subscale, and that this difference in effect size was statistically significant (*z-score* = 2.2, *p* = .026) [64]. Regarding happiness-pursuit and costly prosociality, zero-order correlations revealed that the search for happiness scale was not significantly related to costly prosociality (*r*s = .05–.12, *p*s > .05). However, valuing happiness was significantly related to the self-sacrifice (footbridge) measure and costly prosociality subscale (*r*s = .20-.21, *p*s < .005), and prioritizing positivity was significantly related to all three prosociality measures (*r*s = .19-.29, *p*s < .009).

**Table 5:** Correlations matrix and descriptives in Study 3.

Variable	*M* (*SD*)	1	2	3	4	5	6	7	8	9	10	11	12	13
1. Search for meaning	4.19 (1.76)													
2. Presence of meaning	4.61 (1.62)	**-.28** **												
3. Search for happiness	5.42 (1.33)	**.39** **	-.02											
4. Valuing happiness	4.11 (1.26)	**.51** **	**-.19** **	**.46** **										
5. Prioritising positivity	6.24 (1.59)	**.26** **	**.24** **	**.54** **	**.50** **									
6. Life satisfaction	4.15 (1.67)	-.04	**.58** **	.03	-.02	**.30** **								
7. Kidney donation	3.47 (1.96)	**.22** **	.13	.05	.10	**.19** **	**.23** **							
8. Self-sacrifice (footbridge)	3.59 (2.25)	**.28** **	.11	.12	**.21** **	**.29** **	**.26** **	**.59** **						
9. High-cost prosociality ss	2.95 (1.00)	**.29** **	.08	.11	**.20** **	**.23** **	**.19** **	**.63** **	**.42** **					
10. Low-cost prosociality ss	4.10 (0.80)	.13	.04	**.15** *	-.05	.13	.06	**.31** **	**.22** **	**.47** **				
11. Age	-	**-.26** **	.14	**-.17** ^*^	**-.34** **	-.13	.06	-.08	**-.22** **	-.07	**.25** **			
12. Female	-	-.02	.08	.01	-.05	.01	.05	.05	.00	.11	**.22** **	.06		
13. Conservatism (economic)	3.43 (1.89)	.06	**.30** **	-.01	.02	.08	**.38** **	.00	.10	.05	-.06	**.17** *	-.13	
14. Conservatism (social)	2.95 (1.80)	.03	**.22** **	-.04	.03	.03	**.29** **	.00	.08	-.01	**-.15** *	.09	-.13	**.79** **

*N* = 195

* = *p* < .05

** = *p* < .01; ss = subscale.

As in Study 2, we further tested our hypothesis using multiple regressions predicting each prosociality variable with search for meaning, while including presence of meaning, happiness-pursuit, life satisfaction, and demographic variables as co-variates (Table 6). These regressions revealed that the search for meaning remained a significant predictor of costly prosociality (βs .25–.29, *p*s < .004), further supporting our hypothesis. The positive relationships between happiness-pursuit variables and costly prosociality observed at the zero-order level became non-significant in the regressions (search for happiness: βs = -.10 –-.12, *p*s > .05; valuing happiness: βs = -.04 –.07, *p*s > .05), with the exception of the relationship between prioritizing positivity and self-sacrifice (footbridge; β = .20, *p* = .032). Presence of meaning did not significantly predict costly prosociality in the regressions, while life satisfaction significantly predicted kidney donation and self-sacrifice (footbridge), but not the high-cost prosociality subscale.

**Table 6:** Multiple regressions predicting costly prosociality with search for meaning in Study 3.

Variable	Kidney donation		Self-sacrifice (footbridge)		High-cost prosociality subscale		Low-cost prosociality subscale	
	*Β*	*95% CI*	*β*	*95% CI*	*β*	*95% CI*	*β*	*95% CI*
(Intercept)	.00	-.13, .13	.00	-.13, .13	.00	-.13, .13	.00	-.13, .13
Search for meaning	**.29** **	.12, .46	**.25** **	.09, .41	**.29** **	.12, .45	**.22** **	.06, .38
Presence of meaning	.09	-.09, .28	.03	-.15, .20	.05	-.13, .24	.00	-.18, .18
Search for happiness	-.12	-.29, .05	-.10	-.27, .06	-.10	-.27, .07	.12	-.04, .29
Valuing happiness	-.04	-.23, .14	-.02	-.20, .16	.07	-.11, .26	-.16	-.33, .02
Prioritising positivity	.12	-.07, .31	**.20** *	.01, .38	.11	-.08, .29	.10	-.08, .29
Life satisfaction	**.20** ^*^	.02, .38	**.21** *	.04, .38	.16	-.02, .33	.06	-.11, .23
Age	-.03	-.18, .12	**-.1** * ^*^	-.31, -.02	.00	-.14, .15	**.29** **	.15, .43
Female	.03	-.11, .17	.00	-.13, .13	.10	-.03, .24	**.17** *	.04, .31
Conservatism (economic)	-.15	-.39, .08	-.01	-.23, .22	.05	-.18, .28	.01	-.21, .23
Conservatism (social)	.03	-.19, .26	.02	-.19, .23	-.12	-.34, .10	-.18	-.39, .03

*N* = 195; β = standardized beta; CI = confidence interval

* = *p* < .05

** = *p* < .01; *R*^2^: Kidney donation = .14, Self-sacrifice (footbridge) = .20, High-cost prosociality = .16, Low-cost prosociality = .21.

Study 3 sought to replicate the relationship between the search for meaning and costly prosociality, test if search for meaning related differently to low-cost prosociality, and compare meaning-seeking with pursuing happiness. Consistent with Study 1, and to a lesser extent Study 2, Study 3 revealed consistent positive relationships between the search for meaning and all measures of costly prosociality. Further, results showed that the search for meaning more strongly predicted wanting to perform high-cost rather than low-cost prosocial acts. This finding suggests that meaning-seekers may be more motivated to perform costlier prosocial acts because these acts are viewed as more meaningful [43, 44].

Regarding happiness-pursuit and costly prosociality, our findings were mixed. While two measures of happiness pursuit were significantly related to costly prosociality at the zero-order level (valuing happiness and prioritising positivity), results from the regression analyses show that only prioritizing positivity remained significantly related to costly prosociality, and only for the self-sacrifice (footbridge) measure. Our findings show that when the shared variance between seeking meaning and seeking happiness is controlled for, the relationship between pursuing happiness and costly prosociality generally becomes non-significant. While these results do not suggest that pursuing happiness significantly *decreases* a person’s motivation toward costly prosociality, they do suggest that pursuing happiness is not associated with costly prosocial motivation in the way that meaning-seeking is. However, the significant relationship we observed between prioritizing positivity and self-sacrifice (footbridge) may also indicate that there are some forms of happiness-pursuit which are more likely to motivate costly prosociality; specifically those which are less directly focused on immediate hedonic goals [61].

## Study 4

Extending our earlier studies, Study 4 sought to control for two major aspects of personality that have been previously related to both meaning-seeking and prosocial behavior. Both Big-5 traits Agreeableness and Open-mindedness have been shown to relate to search for meaning [57, 65], and both dimensions often predict prosociality [66, 67]. Therefore, it may be the case that these traits account for the relationship between meaning-seeking and prosociality. To test for this possibility, we controlled for these variables when testing the search for meaning—costly prosociality relationship.

### Method

#### Participants and procedure

Two-hundred and nine American participants completed the study on MTurk in exchange for (US) $1.25. Twenty cases were removed using the same exclusion criteria as in Study 2 and 3, leaving a final sample of 189 participants (100 male, 89 female), aged between 19 and 71 years (*M*_*Age*_ = 35.0, *SD*_*Age*_ = 11.2).

#### Measures

Study 4 retained measures of search for meaning (α = .97), kidney donation (α = .94), footbridge dilemma, and both high-cost (α = .80) and low-cost (α = .71) prosociality from Study 3. Unlike previous studies, we did not measure and control for presence of meaning. Because Study 3 showed robust relationships between search for meaning and costly prosociality at the zero-order level, we did not believe that presence of meaning would mask the relationship between search for meaning and costly prosociality. New, added variables included Big-5 personality measures.

*Big-five dimensions*. We measured Big-5 dimensions using the Big five inventory 2 short-form [68]. Scales for Open-mindedness (e.g., “*Is complex*, *a deep thinker*”; α = .79) and Agreeableness (e.g., “*Is compassionate*, *has a soft heart*”; α = .75) were measured with six items each on a 5-point scale (*1 = disagree strongly; 5 = agree strongly*).

### Results and discussion

Table 7 displays correlations between variables in Study 4. Observed correlations replicate our previous findings, revealing significant positive relationships between the search for meaning and all costly prosociality measures (*r*s = .19–.28, *p*s < .008). Further, search for meaning was again less strongly related to the low-cost prosociality subscale compared to high-cost prosociality. This difference in effect size was only marginally significant (*z-score* = 1.83, *p* = .07). However, when collapsing across Study 3 and Study 4 (*N* = 384), search for meaning had a significantly stronger relationship to the high-cost prosociality subscale compared to the low-cost prosociality subscale (*z-score* = 3.06, *p* = .002).

**Table 7:** Correlations matrix and descriptives in Study 4.

Variable	*M* (*SD*)	1	2	3	4	5	6	7	8	9	10
1. Search for meaning	4.81 (1.72)										
2. Openness	3.77 (0.83)	.14									
3. Agreeableness	3.56 (0.79)	.06	**.48** **								
4. Kidney donation	3.65 (1.83)	**.24** **	-.14	-.02							
5. Self-sacrifice (footbridge)	4.00 (2.12)	**.19** **	**-.15** *	-.07	**.44** **						
6. High-cost prosociality ss	3.04 (1.02)	**.28** **	-.06	.11	**.58** **	**.37** **					
7. Low-cost prosociality ss	4.08 (0.67)	.13	**.25** **	**.30** **	.14	.07	**.33** **				
8. Age		-.09	.03	**.23** **	-.09	-.14	.01	**.14** *			
9. Female		.03	.13	.09	.01	-.12	.13	**.25** **	.09		
10. Conservativism (economic)	3.83 (1.80)	.04	**-.19** **	-.07	.10	**.16** *	.07	-.14	.10	**-.15** *	
11. Conservativism (social)	3.43 (1.82)	.05	**-.25** **	-.12	**.18** ^*^	**.19** **	.14	**-.19** **	.13	**-.17** *	**.80** **

*N* = 189

* = *p* < .05

** = *p* < .01; ss = sub-scale.

Results from multiple regressions (Table 8) further show that the search for meaning predicts costly prosociality when controlling for Agreeableness, Open-mindedness, and demographic variables (βs = .19–.28, *p*s < .009). Regarding other variables, the regressions unexpectedly revealed that Agreeableness was only significantly positively related to one of the costly prosociality measures, and that Open-mindedness was significantly *negatively* related to costly prosociality. Being female predicted the high- and low-cost prosociality subscales, being socially conservative significantly predicted willingness to donate a kidney, and age was negatively associated with self-sacrifice (footbridge).

**Table 8:** Multiple regressions predicting costly prosociality with search for meaning in Study 4.

Variable	Kidney donation		Self-sacrifice (footbridge)		High-cost prosociality subscale		Low-cost prosociality subscale	
	*Β*	*95% CI*	*β*	*95% CI*	*β*	*95% CI*	*β*	*95% CI*
(Intercept)	.00	-.13, .13	.00	-.13, .13	.00	-.13, .13	.00	-.13, .13
Search for meaning	**.23** **	.09, .37	**.19** **	.05, .33	**.28** **	.14, .42	.12	-.02, .25
Agreeableness	.10	-.07, .26	.04	-.12, .20	**.19** *	.03, .35	**.19** *	.04, .35
Open-mindedness	**-.18** ^*^	-.34, -.02	-.14	-.31, .02	**-.17** *	-.33, -.01	.08	-.07, .24
Age	-.11	-.26, .03	-.14	-.29, .00	-.04	-.18, .11	.11	-.03, .25
Female	.05	-.09, .19	-.07	-.21, -.07	**.15** *	.01, .29	**.18** **	.05, .32
Conservatism (economic)	-.14	-.37, .09	.03	-.20, .26	-.11	-.33, .12	.03	-.19, .26
Conservatism (social)	**.27** ^*^	.04, .51	.13	-.11, .37	.22	-.01, .45	-.17	-.39, .06

*N* = 189; β = standardized beta; CI = confidence interval

* = *p* < .05

** = *p* < .01; *R*^2^: Kidney donation = .13, Self-sacrifice (footbridge) = .11, High-cost prosociality = .15, Low-cost prosociality = .18.

Study 4 sought to replicate the relationship between search for meaning and costly prosociality while controlling for Agreeableness and Open-mindedness. Correlational results showed that search for meaning was again significantly related to all measures of costly prosociality, replicating our previous findings. We also found that across Studies 3 and 4 search for meaning had a significantly stronger relationship to costly compared to less costly prosociality. Further to this, results from multiple regressions reveal that the relationship between search for meaning and costly prosociality remains significant when controlling for Agreeableness and Open-mindedness. This provides some evidence that meaning-seeking is a ‘unique’ predictor of costly prosociality, and its relationship to prosociality is not explained by other major personality traits that have been previously linked with meaning-seeking and prosociality.

The results from Studies 1–4 reveal a replicable relationship between searching for meaning and the motivation to engage in costly prosocial acts. While this relationship was observed across a variety of costly prosocial measures, these only measured *intention* to act prosocially. In Study 5, we sought to build on this by examining if the search for meaning was related to enactment costly prosocial behaviors in everyday life.

## Study 5

Study 5 sought to extend previous studies by measuring reported costly prosocial behavior in everyday life, and was preregistered with the Open Science Framework (https://osf.io/vsr4j). As Studies 1–4 revealed a consistent relationship between search for meaning and *motivation* to enact costly prosocial behaviors, we expected that meaning-seekers would also be more likely to report having recently enacted costly prosocial behaviors. Search for meaning is an individual difference variable that can remain stable over longer periods of time [69], and even as presence of meaning increases over time, this does not necessarily reduce the motivation to search for more meaning in the future [65]. Together, these findings suggest that a measure of *current* search for meaning can be used to predict *previous* prosocial behaviors. However, to reduce potential noise due to within-person changes in meaning-seeking over time, we only asked participants about their *recent* prosocial behavior (i.e., in the past three months). We hypothesized that higher search for meaning would predict greater engagement in costly prosocial behaviors in the recent past.

Study 5 further included several new control variables. Both religiosity and Honesty-humility were controlled for as potentially competing predictors of prosociality. Religiosity has been found to be associated with both meaning-seeking [1, 21] and prosociality [70, 71], while Honesty-humility is a trait that roughly captures the “morally-relevant” aspect of personality [72, 73]. We controlled for both variables to ensure that the relationship between meaning-seeking and prosocial engagement was not reducible to merely having religious or virtuous character. Last, to account for the tendency to over-report past prosocial behavior, we measured and controlled for social desirability.

To adequately measure incidence of costly prosocial behaviors, we formed a novel prosociality inventory. The inventory was adapted from previous work measuring prosociality [74] and included 17 different items capturing a broad range of costly prosocial behaviors that individuals may engage in (Table 9). A description of the method for developing the inventory is included in the supporting information (S1 File).

**Table 9:** Prosociality inventory in Study 5.

*No*.	*Behavior*
1	Let someone stay at your house/residence who needed a place to stay.
2	Personally gave money or resources (food, items, etc.) to a stranger or acquaintance in need.
3	‘Babysitted’ someone’s child or pet for no pay.
4	Offered to assist a co-worker or fellow student with a project/assignment/homework, etc.
5	Offered to teach someone a new skill or mentor them in a role.
6	Did a difficult task/chore on someone’s behalf (e.g. mowing neighbor’s lawn, helped move heavy objects or furniture).
7	Took care of a sick or elderly person you know.
8	Volunteered doing fundraising for a charity (e.g. advertising, funrun, etc.).
9	Volunteered in the community (church, school, sports team, clubs, road crossing, etc.).
10	Volunteered to help sick or disaffected people (e.g. at hospital, homeless shelter, soup kitchen, senior home).
11	Donated to local food bank.
12	Donated your own money to a charity/charitable cause.
13	Gave up an object for someone else that you wanted to keep (e.g. food, a cherished item or piece of clothing, etc.).
14	Stopped to help an injured or ailing person.
15	Put myself in danger to help an endangered person (e.g. save someone from drowning, car accident, a violent offender, etc.).
16	Donated blood.
17	Donated an organ.

For each item, participants were asked if they had performed the behavior in the past three months, and if so, the number of times they had performed the behavior in this period.

### Method

#### Participants and procedure

Given the potential noise that can result from asking participants to recall behaviors over the past three months, we recruited a larger sample for Study 5. A power analysis run on G*Power (version 3.0) revealed that to detect a small effect size (*r* = .15) with 0.8 power, we would require a sample of at least 346 participants. To ensure this sample size requirement would be met, 404 US participants completed the survey on MTurk. Following our pre-registered exclusion criteria, 34 cases were removed, leaving a final sample of 370 participants (219 male, 150 female, 1 non-binary). Of this sample, ages ranged 19 to 71 years (*M*_*Age*_ = 35.7, *SD*_*Age*_ = 10.6), and 131 (35.4%) described themselves as religious (120 Christian; 2 Muslim; 2 Buddhist; 1 Jewish; 2 other; 4 non-disclosed).

Participants completed the same measures of search for meaning (α = .97), presence of meaning (α = .94) and demographics as in Studies 1–3, as well as measures of religiosity, social desirability, Honesty-humility and the prosociality inventory.

#### Measures

*Honesty-humility*. We measured Honesty-humility using a 10-item scale (α = .84) derived from the 60-item HEXACO-PI-R [75]. Honesty-humility represents a ‘moral’ or ‘virtuous’ component of personality (e.g., “*I wouldn’t use flattery to get a raise or promotion at work*, *even if I thought it would succeed*”). Participants responded to items using a 5-point scale (*1 = strongly disagree; 5 = strongly agree*).

*Social desirability*. Social desirability was measured using a 10-item version of the Marlowe-Crowne Social Desirability Scale [76]. Participants responded to each item (e.g., “*I’m always willing to admit it when I made a mistake*”) on a 5-point scale (*1 = strongly disagree; 5 = strongly agree*; α = .82)

*Religiosity*. Religiosity was measured using a single item (“*Are you religious*?”) to which participants could indicate ‘yes’ or ‘no’. Participants who indicated ‘yes’ were asked to specify which religion they were affiliated with.

*Prosociality inventory*. Participants were presented with the 17-item costly prosociality inventory (Table 9). When presented with each behavior, participants were asked to indicate whether or not they had engaged in the behavior in the past three months (*0 = No; 1 = Yes*). If ‘yes’ was selected, participants were asked to further indicate the number of times they had performed the behavior within this period on a 10-point scale (*1 = 1 time; 10 = 10+ times*). In the case of each behavior, participants also indicated on a 7-point scale how “costly, demanding, or difficult” they believed the behavior was to perform in general (*1 = not at all costly; 7 = extremely costly*).

Two measures of prosociality were derived from inventory responses. Measure 1 (named ‘Prosociality-categorical’) represented the *total number of ‘kinds’ or ‘categories’ of prosocial behaviors* the participant reported their engagement in (i.e., said ‘yes’ to having performed). Measure 2 (named ‘Prosociality-cumulative’) represented the *total cumulative number of prosocial behaviors* that the participant had reported their engagement in (i.e., for the items/behaviors the participant had said ‘yes’ to, how many times had they performed that behavior in the time-period). The final two versions of the variables were used in tandem to represent costly prosociality in all analyses, and were strongly correlated (*r* = .78, *p* < .001).

### Results and discussion

#### Preliminary analyses

Both the Prosociality-categorical and Prosociality-cumulative measures showed a positive skew with skewness in the Prosociality-cumulative measure being particularly high (2.9). Following our pre-registration, we excluded participant scores from analyses if they scored beyond three standard deviations of the mean on either variable. This led to 12 cases being excluded for the Prosociality-cumulative measure. This exclusion measure reduced the skew in the Prosociality-cumulative measure to 1.3. Removing the cases did not significantly change the nature of the relationships between prosociality and other key variables.

#### Primary analyses

Table 10 presents correlations between variables included in Study 5. Correlations reveal that search for meaning was again negatively related to age and presence of meaning. Search for meaning was also positively associated with religiosity, though surprisingly negatively associated with Honesty-humility and social desirability. Regarding prosociality, zero-order correlations revealed that search for meaning was significantly related to Prosociality-categorical (*r* = .14, *p* = .007), but only marginally related to Prosociality-cumulative (*r* = .10, *p* = .062). Results showed that both religiosity and presence of meaning were significantly associated with having performed costly prosocial behaviors, however, Honesty-humility was surprisingly unrelated to prosociality.

**Table 10:** Correlations matrix and descriptives in Study 5.

Variable	*M* (*SD*)	1	2	4	5	7	8	9	10	11	12
1. Search for meaning	4.43 (1.77)										
2. Presence of meaning	4.72 (1.68)	**-.28** **									
4. Honesty-humility	3.45 (0.84)	**-.26** **	.10								
5. Social desirability	3.00 (0.79)	**-.14** **	**.17** **	**.49** **							
7. Prosociality-categorical	4.38 (3.49)	**.14** **	**.19** **	**-.11** ^*^	-.04						
8. Prosociality-cumulative	13.59 (12.48)	.10	**.18** **	-.03	-.06	**.78** **					
9. Religiosity		**.11** ^*^	**.24** **	.03	.06	**.29** **	**.19** **				
10. Age		**-.17** **	**.14** **	**.13** ^*^	-.02	-.04	.05	.05			
11. Female		-.00	.01	.09	.01	.10	.08	.02	**.12** ^*^		
12. Conservatism (economic)	3.62 (1.92)	-.07	**.21** **	.00	.04	**.14** **	**.13** ^*^	**.29** **	.10	-.09	
13. Conservatism (social)	3.14 (1.89)	-.09	**.26** **	.01	.09	**.15** **	**.12** ^*^	**.37** **	.07	-.04	**.79** **

*N* = 370 (*N* = 358 for Prosociality-cumulative due to outlier removal)

* = *p* < .05

** = *p* < .01.

To further investigate, we ran multiple regressions predicting each prosociality variable with search for meaning, while adding presence of meaning, social desirability, Honesty-humility, and demographic variables (including religiosity) as co-variates (Table 11). Results from these regressions reveal that search for meaning significantly predicted both prosociality measures (βs = .13-.15, *p*s < .02). The regressions also revealed that both presence of meaning and religiosity significantly predicted both prosociality variables, and that being female predicted Prosociality-categorical.

**Table 11:** Multiple regressions predicting costly prosociality with search for meaning in Study 5.

Variable	Prosociality-categorical		Prosociality-cumulative	
	*β*	*95% CI*	*β*	*95% CI*
(Intercept)	.00	-.10, .10	.00	-.10, .10
Search for meaning	**.13** *	.03, .24	**.14** *	.03, .25
Presence of meaning	**.19** **	.13, .35	**.19** **	.07, .30
Social desirability	-.08	-.19, .04	-.08	-.20, .04
Honesty-humility	-.08	-.20, .03	.00	-.12, .12
Religiosity	**.22** **	.11, .32	**.12** *	.00, .23
Age	-.06	-.16, .03	.02	-.09, .12
Female	**.11** *	.02, .21	.09	-.01, .19
Conservatism (economic)	.09	-.07, .24	.11	-.05, .28
Conservatism (social)	-.03	-.19, .14	-.03	-.20, .14

*N* for Prosociality-categorical = 370; *N* for Prosociality-cumulative = 358; β = standardized beta; CI = confidence interval

* = *p* < .05

** = *p* < .01; *R*^2^: Prosociality-categorical = .15, Prosociality-cumulative = .09.

In Study 5, we extended from our previous work examining prosocial intentions to investigate whether the search for meaning predicted reporting real-world engagement in costly prosocial behaviors. Results supported the hypothesized relationship between search for meaning and reporting the enactment of costly prosocial behaviors, even when controlling for a host of other variables that have previously been shown to relate to prosociality. While the effect size was smaller compared to previous studies, this may be explained by i) the noise inherent in a measure that requires participants to accurately recall behaviors they performed up to three months ago, and ii) that the study measured *current* search for meaning but concerned *past* (albeit recent) prosocial behavior. Despite these limitations, and the correlational nature of the results, our findings suggest that meaning-seeking is linked with the enactment of costly prosocial behavior, rather than just intentions toward costly prosociality. Findings also revealed a stronger relationship between presence of meaning and costly prosociality than was found in Studies 1–3. This increase in effect size may partly be due to engagement in past prosocial activity leading to an increase in present sense of meaning [2, 3]. Cross-study meta-analysis: Studies 1–5

**Fig 1:**
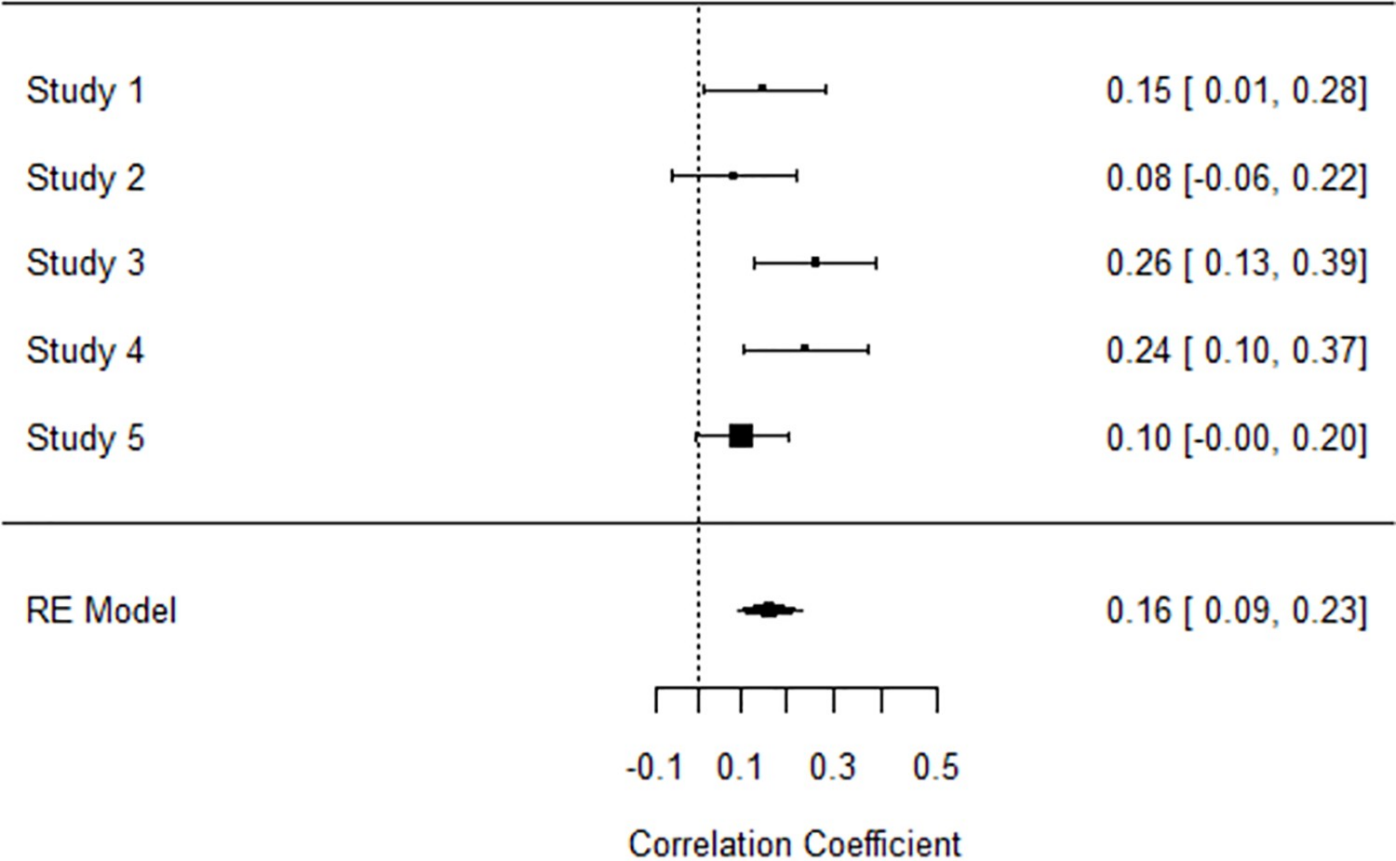
Forest plot of random-effects model for relationship between search for meaning and costly prosociality in Studies 1–5. *N* = 1129.

To address the smaller sample sizes used in Studies 1–4, we conducted a meta-analysis of the search for meaning—costly prosociality relationship across Studies 1–5 using the *robumeta*, *metafor*, and *dplyr* packages in Rstudio [77]. Within each study, we had multiple dependent effects (i.e., correlations between search for meaning and costly prosociality) due to using varied operationalizations of costly prosociality. To amend this for meta-analysis, we aggregated the correlations within each study between search for meaning and each operationalization of costly prosociality, to obtain one correlation per study, and then analyzed these five effects (*N* = 1129). We chose a random-effects model to estimate the overall correlation between search for meaning and costly prosociality across Studies 1–5. For the meta-analysis, we converted Pearson’s *r* coefficients to Fisher’s *z* as recommended by Borenstein and colleagues [78], and then converted results back into correlation coefficients for presentation. The random-effects model revealed that overall, search for meaning and costly prosociality shared a small yet significant correlation (*r* = .16, 95% CIs [.09, .24], *z* = 4.35, *p* < .001; Fig 1 for forest plot). We did not find significant heterogeneity in effect size, *Q*(4) = 6.11, *p* = .191, with *I*^*2*^ = 34.6%, indicating a low-to-moderate level of heterogeneity [79].

## General discussion

To date, psychological literature examining meaning in life has largely focused on the benefits of presence of meaning, while study of the positive correlates of seeking meaning has been largely neglected. While search for meaning has previously been shown to have a negative relationship with *personal* well-being, the present studies demonstrate that meaning-seeking is clearly associated with the will to improve the well-being of others. Studies 1–4 reveal a consistent, positive correlation between the search for meaning and motivation to enact a variety of costly prosocial behaviors. Study 5 furthered this by showing that seeking meaning is associated with reported engagement in costly prosocial behaviors.

The present studies were the first to show how the search for meaning is related to costly prosociality and build an argument for why this is the case. Our theorizing and findings compliment recent work showing how search for meaning is related to general ‘heroism motivation’ [48], and how the need for meaning is related to community-oriented goals and action [55]. While the meta-analyzed relationship between search for meaning and costly prosociality overall was not large, we believe our findings are important in furthering understanding of what underlies prosocial behavior motivation, particularly when these behaviors involve personal sacrifice.

Prosociality may be intrinsically meaningful [24]. However, one factor that bolsters the meaningfulness of a prosocial act is the costliness of these behaviors for the actor [43, 44]. In Studies 3 and 4, we observed that the search for meaning shared a significantly stronger relationship with the motivation to perform costly prosocial actions, compared to less-costly examples. In line with findings by Olivola and Shafir [43, 44], and work detailing the meaningfulness and costliness of heroic action [46, 47], we interpret this difference as due to the greater perceived meaningfulness of costlier prosocial endeavors. Our results from Study 1 show that meaning-seekers are somewhat more likely to view a costly prosocial act as meaningful, but this may not be so much the case for less costly prosocial acts.

### Searching for meaning versus pursuing happiness

Moving beyond previous work detailing the difference or similarity between the *experience* of meaning and happiness, Study 2 and Study 3 examined differences in the *pursuit* of meaning and the pursuit of happiness regarding how they relate to costly prosociality. We hypothesized that the search for meaning would be linked with costly prosociality, but that pursuing happiness would not be positively correlated to prosociality that is costly for the self.

Across Studies 2 and 3, results revealed that the relationship between the search for meaning and happiness-pursuit variables were moderate in size (average *r* = .46), indicating that the motivations to find meaning and happiness in life are related, but distinct [49]. Our results did not indicate that pursuing happiness was significantly *negatively* related to costly prosociality, however, they did show that pursuing happiness is not positively linked to costly prosociality in the way that meaning-seeking is. Previous studies have explored how pursuing happiness can paradoxically lead to lower personal well-being [60, 80]. The present research extends this by showing that pursuing happiness is also unrelated to increasing the well-being of others, particularly when this requires making some sacrifice of the self.

### Presence of meaning and costly prosociality

While the purpose of the present work was to focus on the search for meaning, we also included presence of meaning in most studies as an important co-variate. Presence of meaning has been shown to predict behaviors which are beneficial for the self (e.g., health-promoting behaviors, goal-pursuit, etc.) [81, 82], and some work has shown that presence of meaning is related to greater care for others [56] and community feelings [55, 57]. While it is clear that prosocial acts can increase presence of meaning [2, 3], how presence of meaning predicts motivation toward costly prosocial behavior is less well-known. Our results showed several positive relationships between presence of meaning and costly prosociality motivation at the zero-order level. However, when accounting for other variables in regressions, presence of meaning generally did not predict motivation toward costly prosociality (Studies 1–3) and was only clearly related to reported engagement in recent costly prosocial acts (Study 5). While it has not been our purpose to compare differences between seeking meaning and having meaning, our data does indicate that the relationship between presence of meaning and costly prosociality is less clear compared to search for meaning.

## Implications

To-date, most work examining the social-behavioral outcomes of meaning-seeking has fallen under *Significance Quest Theory*, which has detailed how meaning-seeking is related to violent political extremism or self-sacrifice for a particular political cause [15, 17, 19, 83]. The present work departs from this literature, by displaying that searching for meaning is also related to more ‘benevolent’ instances of costly prosociality that are not centered on political behavior or in-group favoritism. While our data is cross-sectional in nature, our findings theoretically suggest that meaning-seeking may motivate altruistic tendencies. The contrast between the present work and findings within SQT paints the search for meaning as an interesting motivational construct. A search for meaning may produce motivation toward behaviors which are societally-laudable (e.g., kidney donation), or societally-vilified (e.g., killing innocent civilians for a political cause). While we do not dispute that meaning-seekers can gravitate toward extreme political causes, our findings are important in showing that meaning-seekers can also be drawn toward humanitarian behavior that is not entrenched in a particular political ideology.

## Limitations and future directions

We have theorized that people searching for meaning are more motivated to enact costly prosocial behavior due to its capacity to enhance meaning in life. Our theorizing on the search for meaning—costly prosociality link has been supported by showing that the relationship remains robust when accounting for a number of relevant co-variates. However, the studies presented in this paper are cross-sectional, and we have not provided direct evidence showing that search for meaning *causes* prosociality. While it may be of interest to draw on experimental paradigms to seek causal evidence, it is also doubtful whether a complex trait such as the need to seek existential meaning can be effectively manipulated in an experimental setting. Nonetheless, our interpretation of the current findings is supported by considering the low plausibility of the opposite directional pathway, with previous work showing no effect of prosocial behavior on increasing search for meaning [2]. Although experiencing heightened presence of meaning from behaving prosocially could lead people to search for more meaning in life, we think that this explanation of the results is less parsimonious, given that increases in presence of meaning do not lead to significant increases in search for meaning [65].

The present work has replicated the link between meaning-seeking and costly prosociality using a variety of costly prosociality measures, including motivation-based (Studies 1–4) and reports of recent behavior (Study 5). However, a limitation of our approach has been a reliance on self-report measures rather than on measures of objective behavior. In Study 5, we showed that meaning-seeking was associated with reported enactment of costly prosocial behavior, including when accounting for social desirability. Even so, there remains the possibility that meaning-seekers are more likely to indicate or exaggerate their motivation toward and enactment of prosociality without this manifesting in actual behavior. We believe our studies make a valuable contribution by illustrating the robust association between search for meaning and the willingness to enact costly prosocial actions. However, it will be worthwhile for future work to test if meaning-seeking is related to objective behavioral measures of prosociality (such as charitable donation, formal volunteering, or ‘help-the-confederate’ paradigms).

Finally, it is important to note that the present studies all drew from American MTurk samples. Using MTurk for participant recruitment has become increasingly normative within social and personality psychology research and offers a diversity in participants’ individual differences that is generally absent in psychology undergraduate samples [84]. However, recent work has shown that MTurk can produce low quality data [85]. To address this, we took several steps to ensure good data quality, such as including selection criteria on MTurk, implementing data exclusion procedures, and performing psychometric analyses to test the robustness of our data (see S1 File). However, using MTurk across all studies does limit the generalizability of the present findings. Future work should test if the search for meaning–costly prosociality relationship generalizes to non-online samples. It may also be important for future work to test if the search for meaning–costly prosociality relationship replicates using Eastern samples, given that culture can moderate how search for meaning manifests [14].

## Conclusion

The present studies show that the search for meaning is linked with the motivation toward and reported enactment of prosocial behavior, particularly when these behaviors are costly for the actor. The present work further delineates the search for meaning from the pursuit of happiness, by showing that only meaning-seeking, rather than happiness-seeking, is associated with costly prosociality. Ultimately, these findings move beyond previous work that has largely focused on the relationship between meaning and personal well-being–feeling good–and shed light on how the search for meaning in life is linked with striving to improve the well-being of others–doing good.

## Supporting information

S1 File Supplementary materials. (DOCX)
