# Supplementary material for: Searching for meaning is associated with costly prosociality
Source: PLoS One. 2021 Oct 25;16(10):e0258769. doi: 10.1371/journal.pone.0258769 (PMC8544877; doi:10.1371/journal.pone.0258769)
Supplement: S1 File — (DOCX) [file pone.0258769.s001.docx]

# S1 file – Supplementary materials – Search for meaning and costly prosociality

Full description of data robustness and exclusion procedure

- Table A. Data collection date and data exclusion per study
- Attention checks used in each study

Indicators of data quality

- Confirmatory factor analyses
- Table B. Confirmatory factor analyses

Study 3 & 4

- Additional variables
- Table C. Correlations Matrix and Descriptives in Study 3 including SFGM and need for meaning variables.
- Table D. Correlations Matrix and Descriptives in Study 4 including SFGM, need for meaning, and all B5 variables.

Study 5

- Additional variables
- Prosocial inventory development
- Prosocial inventory items – ranked costliness
- Table E. Ranked costliness of each prosocial behavior by participant rating scores
- Honesty humility as moderator variable
- Table F. Testing Honesty-humility as a moderator
- Table G. Correlations matrix for all Study 5 variables including need for meaning and intrinsic religiosity

Miscellaneous

- Presence of meaning as moderator variable
- Table H. Testing presence of meaning as a moderator

# Full description of data robustness and exclusion procedure

Over the five studies, a number of steps were taken to ensure that data used in analyses was of good quality. First, across all studies, we only recruited MTurk workers who had i) completed at least 100 HITs previously, and ii) who had achieved an approval rating of 95% or higher on their previous HITs. Second, we took steps during the data cleaning process to ensure ‘data validity’ and remove any poor-quality responses, which we detail below. The number of responses removed as a result of each of these steps is included below in Table A.

- Across all studies, we removed responses that completed the study in less than one-third of the median completion time, as responses submitted this quickly were likely not reading and answering thoughtfully.
- Across Studies 2-5, we included between one and three attention check questions in each survey (see ‘Attention checks used in each study’ section below). Participants who failed any of these attention checks had their data excluded. An attention check measure was not included in Study 1 due to an oversight.
- **Across Studies 2-4, we looked at participant responses on the ‘qualitative’ response variable for the ‘self-sacrifice (footbridge)’ question where they were asked to write down a group that they belonged to (other than their family). We identified responses where participants had left a suspicious response (e.g. “good”, “family”, etc.), and removed these from the data set prior to analyses.

**This final data-cleaning procedure was done ‘post-hoc’ after the handling editor asked us if we could take more measures to ensure data integrity. This data cleaning procedure, and the confirmatory factor analyses reported below were pre-registered on Aspredicted <https://aspredicted.org/blind.php?x=kk3an2>.

**Table A**

Data collection date and data exclusion per study

| Study | Data collection date | Original *N* | Cases removed: <1/3 MCT | Cases removed: Attention check(s) | Cases removed: suspicious qualitative response | Final *N* |
| --- | --- | --- | --- | --- | --- | --- |
| 1 | 18^th^ Dec 2017 | 210 | 14 | N/A | N/A | 196 |
| 2 | 3^rd^ Apr 2018 | 208 | 7 | 9 | 1 | 191 |
| 3 | 21^st^ May 2018 | 208 | 6 | 5 | 2 | 195 |
| 4 | 2^nd^ Jul 2018 | 209 | 11 | 3 | 6 | 189 |
| 5 | 7^th^ Jan 2019 | 404 | 5 | 29 | N/A | 370 |

*Note*. MCT = median completion time. Case removal procedure was done step-wise (i.e., there were likely more participants who failed attention checks who had already been removed for completing the study in <1/3 MCT.

## Attention checks used in each study

Study 2 (Two attention checks)

1. “please select ‘somewhat true’”
2. “please select ‘very true for me’”

Study 3 (Two attention checks)

1. “please select ‘somewhat true’”
2. “please select ‘not at all likely’”

Study 4 (One attention check)

1. “please select ‘not at all likely’”

Study 5 (Three attention checks)

1. At start of prosocial inventory, following description “Do you understand the nature of the task you are about the perform?” (yes/no)
2. Within Prosociality inventory, “This is an attention check, please select ‘yes’ and then the number ‘7’”
3. At end of prosociality inventory, “in the previous exercise, I reflected on my behaviors over the past BLANK months” (must select ‘3’ to pass

# Indicators of data quality

Below we report on two key psychometric analyses that can indicate data quality: 1) reliability, and 2) confirmatory factor analyses.

Reliability of scales is one useful indicator of data quality (Chmielewski and Kucker, 2020). All scales used throughout Studies 1-5 showed good to excellent reliability (*range* = .71-.97), with an overall mean alpha score of α =.88 (*SD* = .08). The high reliability of scales used across our studies is one indicator that our data was of good quality.

## Confirmatory factor analyses

In addition to reliability analyses, we ran confirmatory factor analyses on all *validated* scales we employed throughout Studies 1-5. Testing whether the model fit is good for the validated scales we used is a further indication of data quality. As seen below in Table B, all well-validated measures (e.g. MLQ, SWLS) showed good model fit (CFIs >.90) and factor loadings. Of the validated measures we employed, only the creators of the MLQ reported a confirmatory factor analysis in their scale development paper for comparison (Steger et al., 2006). The model fit of the MLQ across all studies is directly comparable to the model fit reported by Steger et al. (2006) in their scale creation paper (they report: CFI = 0.93-0.97; RMSEA = 0.04-0.09; see Table 1 of their paper).

The only times where there are exceptions to good model fit in our data is in short-form scales (e.g., Agreeableness and Openness from BFI-2 short-form; Honesty Humility from HEXACO short-form; etc.). The fact that this arises primarily with the short-form scales suggests that this may be due to the nature of these scales rather than our data.

**Table B**

Confirmatory factor analyses

| **Study** | ***N*** | **Measure** | **Items** | **CFI** | **RMSEA** | **SRMR** | **χ^2^ (df)** | **Std. factor loadings**  **Mean (range)** |
| --- | --- | --- | --- | --- | --- | --- | --- | --- |
| 1 | 196 | MLQ | 10 | 0.98 | .077 | .04 | 73.09 (34) ^***^ | S: .91 (.87-.94)  P: .90 (.82-.94) |
| 2 | 191 | MLQ | 10 | 0.97 | .10 | .037 | 103.45 (34) ^***^ | S: .90 (.86-.92)  P: .90 (.85-.93) |
|  |  | VHS | 7 | 0.85 | .136 | .071 | 63.59 (14) ^***^ | .58 (.34-.69) |
|  |  | PPS | 6 | .97 | .097 | .035 | 25.09 (9) ^**^ | .76 (.71-.85) |
|  |  | SWLS | 5 | .99 | .06 | .01 | 8.45 (5) | .84 (.62-.93) |
| 3 | 195 | MLQ | 10 | .98 | .079 | .042 | 75.32 (34) ^***^ | S: .90 (.89-.91) P: .87 (.71-.93) |
|  |  | VHS | 7 | .95 | .09 | .05 | 37.15 (14) ^**^ | .64 (.50-.79) |
|  |  | PPS | 6 | .97 | .11 | .035 | 31.24 (9) ^***^ | .78 (.70-.84) |
|  |  | SWLS | 5 | .94 | .248 | .034 | 64.92 (5) ^***^ | .86 (.69-.94) |
| 4 | 189 | SFM (MLQ) | 5 | .99 | .08 | .008 | 11.03 (5) | .93 (.92-.94) |
|  |  | Openness (BFI-2-S) | 6 | .79 | .22 | .106 | 90.36 (9) ^***^ | .60 (.30-.88) |
|  |  | Agreeableness (BFI-2-S) | 6 | .88 | .135 | .066 | 40.15 (9) ^***^ | .58 (.27-.75) |
| 5 | 370 | MLQ | 10 | .99 | .036 | .016 | 50.61 (34) ^*^ | S: .94 (.93-.95) P: .89 (.73-.95) |
|  |  | Social desirability (short) | 10 | .86 | .099 | .064 | 162.46 (35) ^***^ | .59 (.43-.72) |
|  |  | Honesty-humility (short) | 10 | .71 | .18 | .11 | 442.40 (35) ^***^ | .56 (.38-.84) |

*Note*. CFI = Comparative fit index; RMSEA = Root mean square error of approximation; SRMR = Standardized root mean square residual; MLQ = Meaning in life questionnaire; VHS = Valuing happiness scale; PPS = Prioritising positivity scale; SWLS = Satisfaction with life scale.

# Study 3 & 4

## Additional variables

In Studies 3-5, we also included measures of the ‘need for meaning’ (Zhang et al., 2016) and an ad-hoc measure of search for *greater* meaning. These measures were highly correlated with search for meaning and generally related to costly prosociality in the same way (see Table C, Table D and Table G).

**Need for meaning.** The need for meaning scale (Zhang et al., 2016; 6 items; α = .91) reflects the need to search for meaning or greater meaning in life (e.g. “I need to seek meaning in my life”), and to live a meaningful life generally (“It is important for me to lead a meaningful life”). Participants responded to all items on a 7-point scale (*1 = not at all true of me; 7 = very true of me*). The need for meaning scale was used in Studies 3, 4 and 5.

**Search for greater meaning**. The search for *greater* meaning (SFGM) scale (5 items; α = .98) was adapted from the search for meaning scale to reflect an individual’s need to seek greater meaning in life. We created this adapted version out of initial concerns that some wording in the search for meaning scale may have reflected or implied low presence of meaning. Items were exactly the same as in the search for meaning scale but with terms such as ‘more’, ‘greater’ or ‘increasingly’ added. (see full materials on our OSF page). The search for greater meaning scale was used in Studies 3 and 4.

**Table C**

Correlations Matrix and Descriptives in Study 3 including SFGM and need for meaning variables.

| Variable | *M* (*SD*) | 1 | 2 | 3 | 4 | 5 | 6 | 7 | 8 | 9 | 10 | 11 | 12 | 13 | 14 | 15 |
| --- | --- | --- | --- | --- | --- | --- | --- | --- | --- | --- | --- | --- | --- | --- | --- | --- |
| 1. Search for meaning | 4.19 (1.76) |  |  |  |  |  |  |  |  |  |  |  |  |  |  |  |
| 2. Presence of meaning | 4.61 (1.62) | **-.28^**^** |  |  |  |  |  |  |  |  |  |  |  |  |  |  |
| 3. SFGM | 4.40 (1.82) | **.88^**^** | -.13 |  |  |  |  |  |  |  |  |  |  |  |  |  |
| 4. Need for meaning | 4.51 (1.53) | **.70^**^** | .06 | **.77^**^** |  |  |  |  |  |  |  |  |  |  |  |  |
| 5. Search for happiness | 5.42 (1.33) | **.39^**^** | -.02 | **.41^**^** | **.40^**^** |  |  |  |  |  |  |  |  |  |  |  |
| 6. Valuing happiness | 4.11 (1.26) | **.51^**^** | **-.19^**^** | **.49^**^** | **.37^**^** | **.46^**^** |  |  |  |  |  |  |  |  |  |  |
| 7. Prioritising positivity | 6.24 (1.59) | **.26^**^** | **.24^**^** | **.35^**^** | **.35^**^** | **.54^**^** | **.50^**^** |  |  |  |  |  |  |  |  |  |
| 8. Life satisfaction | 4.15 (1.67) | -.04 | **.58^**^** | .06 | .12 | .03 | -.02 | **.30^**^** |  |  |  |  |  |  |  |  |
| 9. Kidney donation | 3.47 (1.96) | **.22^**^** | .13 | **.27^**^** | **.33^**^** | .05 | .10 | **.19^**^** | **.23^**^** |  |  |  |  |  |  |  |
| 10. Self-sacrifice (footbridge) | 3.59 (2.25) | **.28^**^** | .11 | **.30^**^** | **.30^**^** | .12 | **.21^**^** | **.29^**^** | **.26^**^** | **.59^**^** |  |  |  |  |  |  |
| 11. High-cost prosociality ss | 2.95 (1.00) | **.29^**^** | .08 | **.31^**^** | **.32^**^** | .11 | **.20^**^** | **.23^**^** | **.19^**^** | **.63^**^** | **.42^**^** |  |  |  |  |  |
| 12. Low-cost prosociality ss | 4.10 (0.80) | .13 | .04 | .12 | **.14^*^** | **.15^*^** | -.05 | .13 | .06 | **.31^**^** | **.22^**^** | **.47^**^** |  |  |  |  |
| 13. Age | - | **-.26^**^** | .14 | **-.27^**^** | **-.15^*^** | **-.17^*^** | **-.34^**^** | -.13 | .06 | -.08 | **-.22^**^** | -.07 | **.25^**^** |  |  |  |
| 14. Female | - | -.02 | .08 | .00 | .06 | .01 | -.05 | .01 | .05 | .05 | .00 | .11 | **.22^**^** | .06 |  |  |
| 15. Conservatism (economic) | 3.43 (1.89) | .06 | **.30^**^** | .07 | **.16^*^** | -.01 | .02 | .08 | **.38^**^** | .00 | .10 | .05 | -.06 | **.17^*^** | **-.13** |  |
| 16. Conservatism (social) | 2.95 (1.80) | .03 | **.22^**^** | .04 | **.16^*^** | -.04 | .03 | .03 | **.29^**^** | .00 | .08 | -.01 | **-.15^*^** | .09 | **-.13** | **.79^**^** |

*Note*. *N* = 195; * = *p* < .05, ** = *p* < .01; SFGM = Search for greater meaning; ss = subscale.

**Table D**

*Correlations Matrix and Descriptives in Study 4 including SFGM, need for meaning, and all B5 variables.*

| Variable | *M* (*SD*) | 1 | 2 | 3 | 4 | 5 | 6 | 7 | 8 | 9 | 10 | 11 | 12 | 13 | 14 | 15 |
| --- | --- | --- | --- | --- | --- | --- | --- | --- | --- | --- | --- | --- | --- | --- | --- | --- |
| 1. Search for meaning | 4.81 (1.72) |  |  |  |  |  |  |  |  |  |  |  |  |  |  |  |
| 2. SFGM | 4.83 (1.71) | **.95^**^** |  |  |  |  |  |  |  |  |  |  |  |  |  |  |
| 3. Need for meaning | 5.16 (1.34) | **.72^**^** | **.73^**^** |  |  |  |  |  |  |  |  |  |  |  |  |  |
| 4. Openness | 3.77 (0.83) | .14 | .14 | **.31^**^** |  |  |  |  |  |  |  |  |  |  |  |  |
| 5. Conscientiousness | 3.60 (0.86) | -.03 | -.03 | **.15^*^** | **.40^**^** |  |  |  |  |  |  |  |  |  |  |  |
| 6. Extraversion | 2.87 (0.85) | .09 | .11 | **.17^*^** | **.23^**^** | **.33^**^** |  |  |  |  |  |  |  |  |  |  |
| 7. Agreeableness | 3.56 (0.79) | .06 | .07 | **.17^*^** | **.48^**^** | **.57^**^** | **.37^**^** |  |  |  |  |  |  |  |  |  |
| 8. Neuroticism | 2.73 (0.99) | **.19^*^** | **.16^*^** | .00 | **-.17^*^** | **-.51^**^** | **-.57^**^** | **-.48^**^** |  |  |  |  |  |  |  |  |
| 9. Kidney donation | 3.65 (1.83) | **.24^**^** | **.21^**^** | **.16^*^** | -.14 | -.07 | .10 | -.02 | .02 |  |  |  |  |  |  |  |
| 10. Self-sacrifice (footbridge) | 4.00 (2.12) | **.19^**^** | **.18^*^** | .05 | **-.15^*^** | -.08 | .06 | -.07 | .05 | **.44^**^** |  |  |  |  |  |  |
| 11. High-cost prosociality ss | 3.04 (1.02) | **.28^**^** | **.25^**^** | **.22^**^** | -.06 | .01 | **.16^*^** | .11 | -.11 | **.58^**^** | **.37^**^** |  |  |  |  |  |
| 12. Low-cost prosociality ss | 4.08 (0.67) | .13 | .11 | .13 | **.25^**^** | **.18^*^** | .06 | **.30^**^** | -.13 | .14 | .07 | **.33^**^** |  |  |  |  |
| 13. Age |  | -.09 | -.07 | .08 | .03 | **.26^**^** | .13 | **.23^**^** | **-.30^**^** | -.09 | -.14 | .01 | **.14^*^** |  |  |  |
| 14. Female |  | .03 | .02 | .02 | .13 | .09 | **-.29^**^** | .09 | **.21^**^** | .01 | -.12 | .13 | **.25^**^** | .09 |  |  |
| 15. Conservativism (economic) | 3.83 (1.80) | .04 | .08 | .13 | **-.19^**^** | .00 | -.02 | -.07 | -.12 | .10 | **.16^*^** | .07 | -.14 | .10 | **-.15^*^** |  |
| 16. Conservativism (social) | 3.43 (1.82) | .05 | .10 | .11 | **-.25^**^** | -.06 | .07 | -.12 | **-.15^*^** | **.18^*^** | **.19^**^** | .14 | **-.19^**^** | .13 | **-.17^*^** | **.80^**^** |

*Note*. *N* = 189; * = *p* <.05, ** = *p* <.01; SFGM = search for greater meaning; ss = sub-scale.

# Study 5

## Additional variables

**Intrinsic religiosity**. In Study 5 we also measured ‘intrinsic religiosity’ (see Table G) which is conceivably another competing predictor of prosociality. However, due to an oversight, the intrinsic religiosity items were only administered to those who indicated that they were religious (*N* = 127). For this reason, we only included religiosity (rather than intrinsic religiosity) as a co-variate in the multiple regressions to preserve statistical power in the models. Intrinsic religiosity was measured using two items which were recently used by Ward and King (2018) to represent this construct. The items were “I try hard to live all my life according to my religious beliefs”, and “My whole approach to life is based on my religion” (α = .86). Participants who had identified themselves as religious were further asked to respond to these items on a 7-point scale (*1 = strongly disagree; 7 = strongly agree*).

## Prosociality inventory development

We used the prosocial behavioral intentions scale created by Baumsteiger and Siegel (2018) as an initial template, which included 20 different kinds of prosocial behaviors people may enact in everyday life. From this scale, we removed eight items which either i) described something not strictly prosocial (e.g. environmental or political behavior), ii) described a behavior already adequately captured by another item, or iii) were too vaguely worded to be useful when asking about past behavior. The remaining 12 items were also re-worded to better describe past behavior rather than intentions of future behavior. Next, using a consensus approach we generated five additional types of prosocial acts that people might engage in with some frequency. This produced a prosociality inventory which included 17 different items and captured a broad range of costly prosocial behaviors that individuals may engage in.

## Prosociality inventory items – ranked costliness

We created measures of each prosociality variable which were weighted by the mean costliness rating of each item (see Table E), such that participants received higher scores for performing costlier behaviors. However, these weighted variables were almost perfectly correlated with the non-weighted versions (*r*s > .99) and did not correlate differently to other variables in the study. Therefore, we have only included the non-weighted scores for ease of interpretation.

We also created a ‘perceptions of costliness’ variable that represented the average ‘perception of costliness’ each participant had across the 17 prosocial behaviors. This variable is included in the correlation matrix below (‘costliness perception’ – Table G).

**Table E**

*Ranked costliness (1 = most costly; 17 = least costly) of each prosocial behavior by participant rating mean scores*

| Rank | Description | *M* | *SD* | Original item no. |
| --- | --- | --- | --- | --- |
| 1 | Donated an organ. | 5.62 | 2.11 | 17 |
| 2 | Put yourself in danger to help an endangered person (e.g. save someone from drowning, car accident, a violent offender, etc.). | 5.01 | 2.18 | 15 |
| 3 | Took care of a sick or elderly person you know | 3.64 | 1.79 | 7 |
| 4 | Volunteered to help sick or disaffected people (e.g. at hospital, homeless shelter, soup kitchen, senior home). | 3.52 | 1.75 | 10 |
| 5 | Let someone stay at your house/residence who needed a place to stay. | 3.49 | 1.83 | 1 |
| 6 | Did a difficult task/chore on someone’s behalf (e.g. mowing neighbour’s lawn, helped move heavy objects or furniture). | 3.37 | 1.56 | 6 |
| 7 | Gave up an object for someone else that you wanted to keep (e.g. food, a cherished item or piece of clothing, etc.). | 3.37 | 1.68 | 13 |
| 8 | Personally gave money or resources (food, items, etc.) to a stranger or acquaintance in need. | 3.30 | 1.62 | 2 |
| 9 | Volunteered doing fundraising for a charity (e.g. advertising, funrun, etc.). | 3.27 | 1.67 | 8 |
| 10 | Offered to teach someone a new skill or mentor them in a role. | 3.25 | 1.61 | 5 |
| 11 | Volunteered in the community (church, school, sports team, clubs, road crossing, etc.). | 3.14 | 1.66 | 9 |
| 12 | Donated your own money to a charity/charitable cause. | 3.09 | 1.54 | 12 |
| 13 | Babysitted' someone's child or pet for no pay. | 3.09 | 1.70 | 3 |
| 14 | Donated blood. | 2.92 | 1.77 | 16 |
| 15 | Offered to assist a co-worker or fellow student with a project/assignment/homework, etc. | 2.86 | 1.41 | 4 |
| 16 | Stopped to help an injured or ailing person. | 2.80 | 1.70 | 14 |
| 17 | Donated to local food bank. | 2.40 | 1.36 | 11 |

*Note*. Range for costliness ratings = 1-7.

## Honesty humility as moderator variable

In our pre-registration document, we included a secondary hypothesis that Honesty-humility would moderate the relationship between search for meaning and costly prosociality. While prosociality is likely perceived as meaningful generally, we hypothesised that this would be especially true of those with who were more virtuous/valued virtue highly. We now view it as more logical to include Honesty-humility as a competing predictor of prosociality, rather than a moderator. Due to this, we have omitted the moderation analysis from the main text of this paper and included it in the supplementary materials. Results from moderated regressions (see Table F below) revealed that, contrary to our hypothesis, Honesty-humility did not significantly moderate the relationship between search for meaning and costly prosociality.

**Table F**

*Testing Honesty-humility as a moderator for the search for meaning—costly prosociality relationship in Study 5*

| **Dependant variable** | **Interaction term *t*-value** | **Interaction term *p*-value** |
| --- | --- | --- |
| Prosociality-categorical | -1.11 | 0.27 |
| Prosociality-cumulative | -1.15 | 0.25 |

*Note. N* for Prosociality-categorical = 370; *N* for Prosociality-cumulative = 358.

**Table G**

Correlations matrix for all Study 5 variables including need for meaning and intrinsic religiosity.

| Variable | *M* (*SD*) | 1 | 2 | 3 | 4 | 5 | 6 | 7 | 8 | 9 | 10 | 11 | 12 | 13 |
| --- | --- | --- | --- | --- | --- | --- | --- | --- | --- | --- | --- | --- | --- | --- |
| 1. Search for meaning | 4.43 (1.77) |  |  |  |  |  |  |  |  |  |  |  |  |  |
| 2. Presence of meaning | 4.72 (1.68) | **-.28^**^** |  |  |  |  |  |  |  |  |  |  |  |  |
| 3. Need for meaning | 5.01 (1.31) | **.64^**^** | **.13^*^** |  |  |  |  |  |  |  |  |  |  |  |
| 4. Honesty-humility | 3.45 (0.84) | **-.26^**^** | .10 | -.10 |  |  |  |  |  |  |  |  |  |  |
| 5. Social desirability | 3.00 (0.79) | **-.14^**^** | **.17^**^** | -.02 | **.49^**^** |  |  |  |  |  |  |  |  |  |
| 6. Intrinsic religiosity | 5.26 (1.44) | -.05 | **.34^**^** | **.22^*^** | **.25^**^** | .16 |  |  |  |  |  |  |  |  |
| 7. Prosociality-categorical | 4.38 (3.49) | **.14^**^** | **.19^**^** | **.16^**^** | **-.11^*^** | -.04 | .01 |  |  |  |  |  |  |  |
| 8. Prosociality-cumulative | 13.59 (12.48) | .10 | **.18^**^** | **.14^**^** | -.03 | -.06 | .08 | **.78^**^** |  |  |  |  |  |  |
| 9. Religiosity |  | **.11^*^** | **.24^**^** | **.25^**^** | .03 | .06 | NA | **.29^**^** | **.19^**^** |  |  |  |  |  |
| 10. Age |  | **-.17^**^** | **.14^**^** | -.01 | **.13^*^** | -.02 | .13 | -.04 | .05 | .05 |  |  |  |  |
| 11. Female |  | -.00 | .01 | .09 | .09 | .01 | .16 | .10 | .08 | .02 | **.12^*^** |  |  |  |
| 12. Conservatism (economic) | 3.62 (1.92) | -.07 | **.21^**^** | **.11^*^** | .00 | .04 | **.21^*^** | **.14^**^** | **.13^*^** | **.29^**^** | .10 | -.09 |  |  |
| 13. Conservatism (social) | 3.14 (1.89) | -.09 | **.26^**^** | **.11^*^** | .01 | .09 | **.28^**^** | **.15^**^** | **.12^*^** | **.37^**^** | .07 | -.04 | **.79^**^** |  |
| 14. Costliness perception | 3.42 (1.16) | .08 | **-.11^*^** | -.03 | **-.14^**^** | -.09 | .01 | **.15^**^** | .03 | .03 | **-.11^*^** | .00 | -.01 | -.04 |

*Note*. *N* = 358 for Prosociality-cumulative, *N* = 127 for Intrinsic religiosity, and *N* = 370 for all other correlations; * = *p* <.05; ** = *p* <.01.

# Miscellaneous

## Presence of meaning as moderator variable

We ran post-hoc exploratory analyses testing if presence of meaning moderated the relationship between search for meaning and costly prosociality. As seen in Table H, presence of meaning was a significant moderator at times, but there was not a consistent pattern of moderation across studies. Ultimately, it is unclear from our findings how one’s level of presence of meaning may affect their search for meaning relating to costly prosociality.

**Table H**

*Testing Presence of meaning as a moderator for the search for meaning—costly prosociality relationship in Studies 1, 2, 3 and 5*

|  | **Dependant variable** | **Interaction term  *t*-value** | **Interaction term  *p*-value** |
| --- | --- | --- | --- |
| Study 1 | Kidney donation | -2.14 | .03 |
|  | Self-sacrifice (entities) | -2.34 | .02 |
| Study 2 | Kidney donation | -0.52 | .60 |
|  | Self-sacrifice (entities) | 1.50 | .14 |
|  | Self-sacrifice (footbridge) | .086 | .39 |
| Study 3 | Kidney donation | -0.08 | .94 |
|  | Self-sacrifice (footbridge) | -1.37 | .17 |
|  | High-cost prosociality subscale | 1.34 | 0.18 |
| Study 5 | Prosociality-categorical | 0.26 | 0.79 |
|  | Prosociality-cumulative | -0.86 | 0.39 |
